# Uncovering differential identifiability in network properties of human brain functional connectomes

**DOI:** 10.1162/netn_a_00140

**Published:** 2020-07-01

**Authors:** Meenusree Rajapandian, Enrico Amico, Kausar Abbas, Mario Ventresca, Joaquín Goñi

**Affiliations:** School of Industrial Engineering, Purdue University, West Lafayette, IN, USA; School of Industrial Engineering, Purdue University, West Lafayette, IN, USA; Purdue Institute of Integrative Neuroscience, West Lafayette, IN, USA; School of Industrial Engineering, Purdue University, West Lafayette, IN, USA; Purdue Institute of Integrative Neuroscience, West Lafayette, IN, USA; School of Industrial Engineering, Purdue University, West Lafayette, IN, USA; School of Industrial Engineering, Purdue University, West Lafayette, IN, USA; Purdue Institute of Integrative Neuroscience, West Lafayette, IN, USA; Weldon School of Biomedical Engineering, West Lafayette, IN, USA

**Keywords:** Brain connectomics, Functional connectivity, Fingerprint, Network science, Subject identifiability

## Abstract

The identifiability framework (𝕀*f*) has been shown to improve differential identifiability (reliability across-sessions and -sites, and differentiability across-subjects) of functional connectomes for a variety of fMRI tasks. But having a robust single session/subject functional connectome is just the starting point to subsequently assess network properties for characterizing properties of integration, segregation, and communicability, among others. Naturally, one wonders whether uncovering identifiability at the connectome level also uncovers identifiability on the derived network properties. This also raises the question of where to apply the 𝕀*f* framework: on the connectivity data or directly on each network measurement? Our work answers these questions by exploring the differential identifiability profiles of network measures when 𝕀*f* is applied (a) on the functional connectomes, and (b) directly on derived network measurements. Results show that improving across-session reliability of functional connectomes (FCs) also improves reliability of derived network measures. We also find that, for specific network properties, application of 𝕀*f* directly on network properties is more effective. Finally, we discover that applying the framework, either way, increases task sensitivity of network properties. At a time when the neuroscientific community is focused on subject-level inferences, this framework is able to uncover FC fingerprints, which propagate to derived network properties.

## Author Summary

Functional connectome (FC) fingerprinting recently became a topic of great interest in network neuroscience. We recently proposed a framework to improve brain fingerprint (i.e., identifiability) of FCs, which improves not only test-retest reliability but also the correlation of FCs with fluid intelligence. However, does this improvement in FC fingerprints propagate to the derived network measures? In this work we found that improving the fingerprint (differential identifiability) of the functional connectome also improves the “fingerprint” of its network properties. Furthermore, when using the identifiability framework on the network properties directly, certain network properties like search information and communicability add to the FC fingerprint. Finally, we show that enhancement of the fingerprint in the network measures, in a wide range of cognitive tasks, using the identifiability framework also improves task sensitivity in these measures. We show that regardless of whether you are using functional connectomes or the network properties derived from them, using the 𝕀*f* framework on the functional connectomes would be a beneficial first step.

## INTRODUCTION

The analysis of structural and functional human brain connectivity based on network science has become prevalent for understanding the underlying mechanisms of the human brain. Using network properties, we are able to understand the topology of brain connectivity patterns (Fornito, Zalesky, & Bullmore, 2016; Sporns, 2010, 2018), integration and segregation (Cohen & D’Esposito, 2016; Deco, Tononi, Boly, & Kringelbach, 2015; Fukushima et al., 2018; Sporns, 2013, Sporns & Betzel, 2016), as well as communication dynamics (Avena-Koenigsberger, Misic, & Sporns, 2018; Costa, Batista, & Ascoli, 2011; Estrada & Hatano, 2008; Petrella, 2011) and association between human cognition and brain function (Alavash, Hilgetag, Thiel, & Gießing, 2015; Bola & Sabel, 2015; Davison et al., 2015; Mattar, Betzel, & Bassett, 2016; Zalesky, Fornito, & Bullmore, 2010). Until recently, many brain connectivity studies used group-level comparisons, where data from many subjects are collapsed (e.g., group averaging) into a representative sample of clinical and healthy population (Castellanos, Di Martino, Craddock, Mehta, & Milham, 2013; Crossley et al., 2014; Fornito, Zalesky, & Breakspear, 2015). However, this comes at a price of potentially ignoring intragroup individual variability (Seitzman et al., 2019).

Detecting individual differences in functional connectivity profiles thus becomes important, when associating connectivity profiles with individual behavioral outcomes. In recent years, publicly available functional connectome (FC) datasets (Biswal et al., 2010; Van Essen et al., 2013) with large sample sizes have enabled the scientific community to account for interindividual variability in the human functional connectome. A number of promising methods that can successfully capture these individual differences have been established in recent times (Gratton et al., 2018; Mars, Passingham, & Jbabdi, 2018; Satterthwaite, Xia, & Bassett, 2018; Seitzman et al., 2019; Venkatesh, Jaja, & Pessoa, 2019). For instance, work by Finn et al. (2015) has shown the existence of a recurrent and reproducible fingerprint in functional connectomes estimated from neuroimaging data. This idea has been extended to maximize or minimize subject-specific and/or task-specific information (Pallarés et al., 2018; Xie et al., 2018). These subject-specific fingerprints have been used to track fluctuations in attention at the individual level (Rosenberg et al., 2019).

The “identifiability framework” (Amico and Goñi, 2018b), based on the group-level principal component analysis of functional connectomes that maximizes differential identifiability, has been shown to improve functional connectome fingerprints within and across sites, for a variety of fMRI tasks, over a wide range of scanning length, and with and without global signal regression (Amico and Goñi, 2018b; Bari, Amico, Vike, Talavage, & Goñi, 2019). Additionally, it has been shown that maximizing differential identifiability on the functional connectomes provides more robust and reliable associations with cognition (Svaldi, Goñi, Abbas, et al., 2019) as well as with disease progression (Svaldi, Goñi, Sanjay, et al., 2019). The natural next step is to assess the impact of such a procedure on subsequent network measurements that characterize topological and communication properties of functional brain networks.

An open question of great relevance for the brain connectomics community is how to measure and uncover subject fingerprints in network measurements of functional connectivity. Uncovering reliable connectivity fingerprints is crucial when assessing clinical populations and when ultimately mapping cognitive characteristics into connectivity (Scheinost et al., 2019; Shen et al., 2017; Svaldi, Goñi, Sanjay, et al., 2019). Our hypothesis is that improvement in FC fingerprints should also “propagate” to network derived measurements. An organic way of assessing this would be to track differential identifiability scores of derived network features as the differential identifiability on the functional connectomes changes. One could also proceed with the application of the identifiability framework directly on the network-derived features as opposed to using it on FCs. The above-mentioned approaches rely on different principles of what a fingerprint in a network-derived measurement. The first one assumes that functional connectivity data are “holding” the fingerprints and propagating them to any network-derived measurement. The second one considers functional connectivity data as a proxies to ultimately estimate a network measurement with a potentially prominent subject fingerprint.

## METHODS

The dataset used here is composed of the 100 unrelated subjects of the Human Connectome Project Release Q3 (Van Essen et al., 2013). Per HCP protocol, all subjects gave written informed consent to the HCP consortium. Each subject consists of two fMRI resting-state runs and seven fMRI tasks: gambling, relational, social, working memory, motor, language, and emotion. Data acquisition for each subject and for each task consists of two fMRI sessions, which are tagged here as test and retest. A cortical parcellation into 360 brain regions as proposed by Glasser et al. (2013) was employed with an additional 14 subcortical regions for completeness (Amico & Goñi, 2018a, 2018b). The HCP functional preprocessing pipeline was used (Glasser et al., 2013; Smith et al., 2013), followed by further processing as described in Amico, Arenas, and Goñi (2019) and Amico and Goñi (2018b) for both resting-state and task fMRI data. For each subject and fMRI session, a symmetric weighted connectivity matrix (the functional connectome) was obtained by computing Pearson’s correlation coefficients between pairs of nodal time courses. For a detailed description of all the preprocessing steps, refer to Amico and Goñi (2018b). Finally, before finding the below network properties, all negative correlations are set to a small value of epsilon (MATLAB command eps, equivalent to 2.22 × 10 − 16). Please note that we used the value of epsilon and not 0 to ensure the following two properties for all FCs assessed: (a) FCs are connected graphs; (b) The derived Markov Chains (as obtained by the transition probability matrices) are regular and hence permit mean first passage time (MFPT) computation (Kemeny & Snell, 1976).

### Network Properties

Graph theoretic measures have played a key role in understanding the attributes of brain networks in general, and of functional connectomes in particular (Fornito et al., 2016; Rubinov & Sporns, 2010; Sporns, 2010). Here we select a set of node and node pair properties (i.e., properties that are a function of a single node or a pair of nodes, respectively) to assess their fingerprinting characteristics. A functional connectome is a symmetric square correlation matrix that may be seen as an undirected weighted graph. Let *G* = (*V*, ***W***) be an undirected weighted graph with set of nodes *V* = {*v*_1_, *v*_2_, …, *v*_*n*_} and weights ***W*** = [*w*_*ij*_], where *w*_*ij*_ is the strength of the edge between nodes *v*_*i*_ and *v*_*j*_.

1.  Degree strength
  The degree strength of a node (*K*_*i*_) in an undirected binary graph is the number of edges that are connected to the node. Here, we consider the weighted sum of the edges connected to the node *i*.
  $$K_{i}=\overset{n}{\underset{j=1}{\sum }}w_{ij}$$
2.  Shortest path length
  The shortest path length (SPL) between two nodes of an undirected graph is defined as the minimum number of edges (and thus steps) that separate the two nodes. For an undirected weighted graph, it is the path that results in the smallest value of the sum of the inverse of edge weights that constitute a path between a pair of nodes *i* and *j*. For such a path, that consists of the following sequence of nodes, Ω_*i*↔*j*_ = {*i*, *x*, *y*, …, *z*, *j*} with corresponding sequence of edge weights *π*_*i*↔*j*_ = {*w*_*ix*_, *w*_*xy*_, …, *w*_*zj*_}, the shortest path length is:
  $$SPL_{ij}=\underset{w_{lm}\in \pi_{i↔j}}{\sum }\frac{1}{w_{lm}}.$$
  Note that Ω_*i*↔*j*_ = Ω_*j*↔*i*_ for shortest paths in any undirected graph.
3.  Search information
  The search information (*SI*_*ij*_) for two nodes *i* and *j* is the information required to follow the shortest path (Rosvall, Trusina, Minnhagen, & Sneppen, 2005); that is, the negative log of the product of probability of taking the correct exit at every node along the shortest path. In other words, it can be considered as the information required to reach node *j* starting from node *i*. For a path between nodes *i* and *j* that has a sequence of nodes Ω_*i*→*j*_ = {*i*, *x*, *y*, …, *z*, *j*}, with probability of taking the path *P*(*π*_*i*→*j*_) = Π_*l*∈$\Omega_{i\to j}^{*}$_1/*k*_*l*_, the search information for the path is (Goñi et al., 2014)
  $$SI_{ij}=-\log_{2}P(\pi_{i\to j}).$$
  Note that *SI*_*ij*_ ≠ *SI*_*ji*_.
4.  Mean first passage time
  The MFPT is the expected (on average) number of steps a random walker takes to reach node *j* (for the the first time) from node *i* (Kemeny & Snell, 1976). The Mean First Passage Time (MFPT) for a pair of nodes with source *i* and target *j* is
  $$MFPT_{ij}=\frac{\zeta_{jj}-\zeta_{ij}}{\varphi_{j}}$$
  where *φ* is the left eigenvector associated with eigenvalue 1, *Z* = [*ζ*_*ij*_] is the fundamental matrix computed as *Z* = (*I* − *P* + Φ)^−1^. Here *I* is the *n* × *n* identity matrix, *P* is the transition matrix and Φ is an *n* × *n* matrix with each column corresponding to the probability vector *φ* such that ∀*j*Φ_*ij*_ = *φ*_*i*_. Please note that *MFPT*_*ij*_ ≠ *MFPT*_*ji*_.
5.  Driftness
  We use a measure of communication called driftness (Costa et al., 2011), which is the ratio of the mean first passage time and the shortest path of a pair of nodes *i* and *j*. Considering that *SP*_*ij*_ is the best possible scenario path for a random-walk, this measurement is modulating the mean first passage times with respect to the fastest routes within the network to go from node *i* to *j*. Hence, note that *W*_*ij*_ ≥ 1.
  $$W_{ij}=\frac{MFPT_{ij}}{SP_{ij}}$$
6.  Communicability
  Communicability between two nodes *i* and *j* is a measure of network integration computed as a weighted sum of number of all possible walks between them. (Estrada & Hatano, 2008). Here, we use a normalization method proposed to handle the disproportionate influence of highly connected nodes (also known as hubs) in a graph (Crofts & Higham, 2009). Note that this is frequently the case when assessing functional connectomes.
  $$C_{ij}={\left[e^{D^{-0.5}AD^{-0.5}}\right]}_{ij}$$
  where *D* = *diag*(*K*) and *K* = [*k*_*i*_] where *k*_*i*_ is the degree strength of node *i*, as defined above.
7.  Clustering Coefficient
  The clustering coefficient of a node is the tendency of its neighbors to form cliques. It is the ratio of the total number of triangles that a node forms with its neighbors to the total number of possible triangles that can be formed.
  $$CC_{i}=\frac{2t_{i}}{k_{i}(k_{i}-1)}$$
  where *t*_*i*_ = 1/2∑_*j*,*h*∈*V*_(*w*_*ij*_*w*_*ih*_*w*_*jh*_)^1/3^ is the geometric mean of triangles around node *i* for weighted networks.
8.  Betweenness Centrality
  The betweenness centrality of a node is the fraction of all shortest paths in a network that contain that node.
  $$B_{i}=\frac{1}{\left(n-1)(n-2\right)}\underset{\begin{array}{c} h,j\in V\\ h\neq j,h\neq i,j\neq i \end{array}}{\sum }\frac{\rho_{hj}(i)}{\rho_{hj}}$$
  where *ρ*_*hj*_(*i*) is the number of shortest paths between *h* and *j* that pass through *i*. It can be seen as a measurement of to what extent a node “lies” between other pairs of nodes when accounting specifically for shortest-paths.

### Group-Level Principal Component Analysis and Differential Identifiability

Briefly describing the Identifiability Framework (𝕀*f*) introduced in Amico and Goñi (2018b), the functional connectomes of each subject (test and retest) are vectorized and added to a matrix, the columns of which are the runs (test and retest) of each subject, while the rows are the functional connectivity values of brain region pairs. The *m* principal components of this matrix are then ranked by variance explained and included, in an iterative fashion, to reconstruct the functional connectomes (Amico and Goñi, 2018b). This is done separately for each task and rest. Following the reconstruction of the functional connectomes, we then compute the network property of interest for each subject, on each run (test and retest). This is referred to as *NP*(𝕀*f*{*FC*}) in all further sections, where *NP* is the network property and *FC* is the functional connectome.

We also extend the framework by using this decomposition — reconstruction procedure on the network properties. In this case, the network properties are computed on the original functional connectomes for each subject and run. Each network property is then vectorized and added to a matrix. Note that this is similar to how functional connectomes were rearranged in the *NP*(𝕀*f*{*FC*}) and in Amico and Goñi (2018b). However, the rows of this matrix now consists of the network property values corresponding to a pair of brain regions in case of pairwise properties or a brain region when node properties are derived. The principal components of this matrix are then extracted and iteratively reconstructed using *m* number of components with the highest explained variance. Since the network properties are the ones being decomposed in this case, the result of the reconstruction is the corresponding network properties of each individual and each run. This method is subsequently referred to as 𝕀*f*{*NP*(*FC*)}).

We use differential identifiability (Amico and Goñi, 2018b) to assess the individual fingerprint of each network property. For each method described above, the network properties derived are used to compute the identifiability matrix. Each position of the identifiability matrix *i*, *j* denotes the correlation between the network property of subject *i* test and subject *j* retest. Then, along the diagonal elements, we have the correlation of a network property between the subject test and retest called *I*_*self*_. The non-diagonal elements are the correlations between a run of a subject *i* and subject *j* where *i* and *j* are different (*I*_*others*_). The differential identifiability is then defined as,

$$I_{diff}=(I_{self}-I_{others})\times 100$$

Intraclass correlation coefficient (ICC) represents how strongly measures of a group are in agreement with each other (Bartko, 1966; McGraw & Wong, 1996). The higher the ICC value, the higher the level of agreement. We use ICC (Shrout & Fleiss, 1979) to assess the task sensitivity of a network measure, for each brain region pair and every subject. In this case, the members of the groups are the different runs (test and retest) of a subject; the different groups represent the different fMRI task conditions (and rest). The mean task sensitivity is then taken across all subjects and reported. For this assessment, the functional connectome (or the network property 𝕀*f*{*NP*(*FC*)}) was optimally reconstructed, that is, using the number of components that gave the highest *I*_*diff*_ score for that task.

## RESULTS

The dataset used for this study consisted of fMRI scans of the 100 unrelated subjects from the Human Connectome Project (Van Essen et al., 2013). For each subject, we computed 18 whole-brain functional connectivity matrices: 4 corresponding to resting-state (2 sessions, each with test and retest), and 14 corresponding to each of the seven tasks (each including two runs; test-retest). The multimodal parcellation used here, as proposed by Glasser et al. (2016), includes 360 cortical brain regions. For completeness, 14 subcortical regions were added (Amico & Goñi, 2018a), hence producing functional connectome matrices (square, symmetric) of size 374 × 374.

In this work, we study the effects of 𝕀*f* on the identifiability profiles of network properties in two different scenarios: (a) when applying differential identifiability on functional connectivity, *NP*(𝕀*f*{*FC*}), and (b) when applying differential identifiability directly on network properties, 𝕀*f*{*NP*(*FC*)}.

*NP*(𝕀*f*{*FC*}): The functional connectomes (FCs) of each task (including rest) were vectorized, organized together, and then decomposed into principal components and subsequently reconstructed by adding an increasing number of components ordered by their variance explained. After every such reconstruction, a number of network measurements (see the Methods section for details) were computed for each FC, and *I*_*diff*_ was found on the derived network properties. This is compared with the *I*_*diff*_ score estimated directly from the reconstructed functional connectomes - 𝕀*f*{*FC*}. By doing so, we extend the differential identifiability framework to uncover fingerprints in network properties derived from functional connectomes.

**Figure 1.**
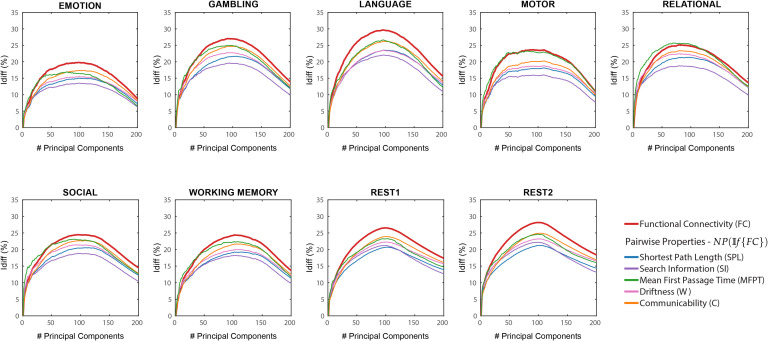
*NP*(𝕀*f*{*FC*}) Differential identifiability (*I*_*diff*_) profiles of pairwise properties for different fMRI tasks as a function of the number of principal components used for reconstruction. Here, the identifiability framework was applied on the functional connectomes (𝕀*f*{*FC*}). Each plot shows, for each fMRI task, the *I*_*diff*_ score associated with functional connectivity *(red solid line)* and the *I*_*diff*_ scores on network properties derived from the reconstructed functional connectomes, *NP*(𝕀*f*{*FC*}) (see legend) for different numbers of components.

For each task, we observed an optimal point of reconstruction where the differential identifiability on the FCs was maximized (see Figure 1). This optimal point was always in the neighborhood of half the maximum number of components (which is equal to the number of subjects in the data) and produced *I*_*diff*_ values much higher than fully reconstructed data, that is, using all the components. These results reaffirm those reported by Amico and Goñi (2018b). We then assessed *I*_*diff*_ on the following node pair network properties: shortest path length (SPL), search information (SI), mean first passage time (MFPT), driftness (W), and communicability (C). In all cases, there was an optimal regime of number of components that maximized *I*_*diff*_ (see Figure 1). Overall, the *I*_*diff*_ score on all the network properties and functional connectomes reach the peak at a similar number of principal components, ranging between 80 and 110. We can also see that the *I*_*diff*_ on functional connectomes is generally higher than those on the network properties for all the tasks and for most of the number of components. One exception is MFPT on motor task where the *I*_*diff*_ scores on FC and MFPT produced very similar results for the entire range of principal components. Another exception is MFPT on relational task where the peak *I*_*diff*_ of *MFPT*(𝕀*f*{*FC*} is greater than that of 𝕀*f*{*FC*} but the margin of difference is really small (≈ 0.59).

**Figure 2.**
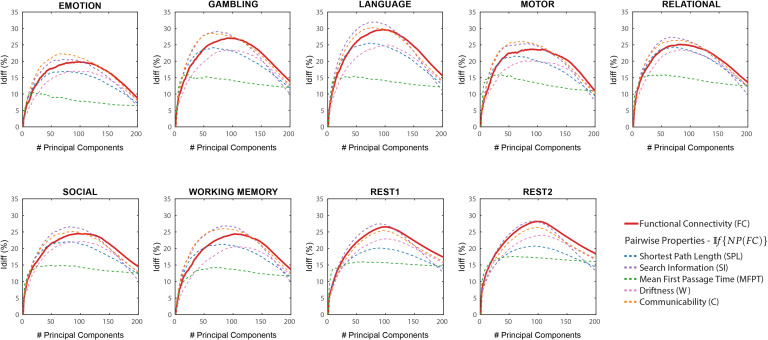
𝕀*f*{*NP*(*FC*)} Differential identifiability (*I*_*diff*_) profiles of pairwise properties for different fMRI tasks as a function of the number of principal components used for reconstruction. Here, the identifiability framework was applied directly on the network properties derived from the original functional connectomes (𝕀*f*{*NP*(*FC*)}). Each plot shows, for each fMRI task, the *I*_*diff*_ score associated with functional connectivity (red solid line) and the *I*_*diff*_ scores on reconstructed network properties derived from the original functional connectomes, 𝕀*f*{*NP*(*FC*)} (see legend) for different numbers of components.

In 𝕀*f*{*NP*(*FC*)}) the different network properties (refer Methods) were first derived from the original functional connectomes and subsequently decomposed and reconstructed using the identifiability framework. *I*_*diff*_ scores were computed on these reconstructed network properties for a different number of components and compared with those computed from the reconstructed FCs (see Figure 2).

**Figure 3.**
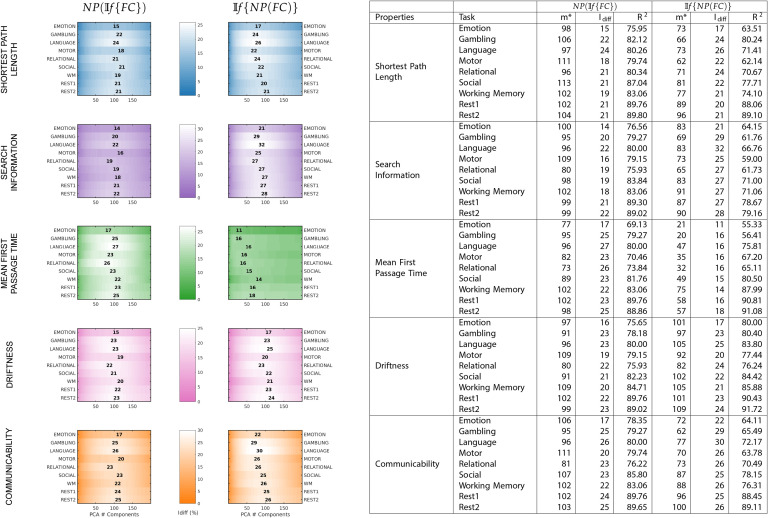
A summary of maximum *I*_*diff*_ values, corresponding to number of components and explained variance retained for each fMRI task and network property for both *NP*(𝕀*f*{*FC*}) and 𝕀*f*{*NP*(*FC*)}. On the left, each plot shows, for each property and each method—*NP*(𝕀*f*{*FC*}) or 𝕀*f*{*NP*(*FC*)}—the *I*_*diff*_ score for all tasks. The number mentioned gives the maximum *I*_*diff*_ score for the corresponding task (y-axis) and the position denotes the number of components (x-axis). On the right is the same information summarized as a table. For each method and network property, the table gives the number of components used for optimal reconstruction m*, corresponding maximum *I*_*diff*_ value, and the variance explained at that reconstruction *R*^2^.

As opposed to results shown in Figure 1, which used *NP*(𝕀*f*{*FC*}), network properties have heterogeneous *I*_*diff*_ profiles with respect to number of components. Compared with *I*_*diff*_ from 𝕀*f*{*FC*}, search information has a higher peak *I*_*diff*_ score for all tasks, while communicability has a higher peak *I*_*diff*_ score for all tasks except resting state. We also find that MFPT has a very different *I*_*diff*_ profile compared with other network properties. The *I*_*diff*_ profiles of MFPT from 𝕀*f*{*MFPT*(*FC*)} increases as we add the first few component and saturates or decreases gradually as more components are added (starting at around 20 components for all tasks). This is unlike other network properties and functional connectomes that share similar *I*_*diff*_ profiles (see Figure 2). A summary of maximum *I*_*diff*_, corresponding number of components used and variance retained for *NP*(𝕀*f*{*FC*}), and 𝕀*f*{*NP*(*FC*)} can be seen in Figure 3.

**Figure 4.**
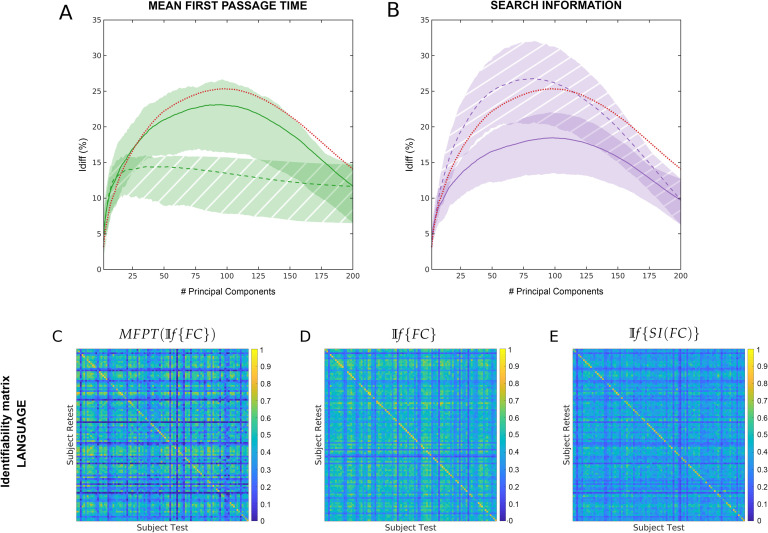
Assessment of the two most divergent network measurement *I*_*diff*_ profiles. (A) Across tasks and rest differential identifiability (*I*_*diff*_) for mean first passage time as a function of the number of principal components used for reconstruction. Solid line and solid shaded area represent the results for *MFPT*(𝕀*f*{*FC*}). Dashed line and hatched area show results for 𝕀*f*{*MFPT*(*FC*)}. (B) Across tasks and rest differential identifiability (*I*_*diff*_) for search information as a function of the number of principal components used for reconstruction. Solid line and solid shaded area represent the results for *SI*(𝕀*f*{*FC*}). Dashed line and hatched area show results for 𝕀*f*{*SI*(*FC*)}. The differential identifiability matrix (as defined in the Methods section) is shown at optimal reconstruction for language task for (C) *MFPT*(𝕀*f*{*FC*}), (D) 𝕀*f*{*FC*} and (E) 𝕀*f*{*SI*(*FC*)}. The diagonal elements in each matrix represent *I*_*self*_ and the non-diagonal elements represent *I*_*others*_.

The network property with the most different *I*_*diff*_ profiles was between *MFPT*(𝕀*f*{*FC*}) and 𝕀*f*{*MFPT*(*FC*)}. Search information was the only network property that reached higher *I*_*diff*_ values for all fMRI tasks for 𝕀*f*{*SI*(*FC*)}. The difference between search information and mean first passage time are assessed in detail in Figure 4. shaded area highlights the variability of *I*_*diff*_ scores across different tasks for *NP*(𝕀*f*{*FC*}) (solid area) and 𝕀*f*{*NP*(*FC*)} (hatched area). Across all tasks, *I*_*diff*_ on 𝕀*f*{*SI*(*FC*)} is higher than *SI*(𝕀*f*{*FC*}).

However, for Mean First Passage time, *I*_*diff*_ on *MFPT*(𝕀*f*{(*FC*)} is higher than (𝕀*f*{*MFPT*(*FC*)}. When *SI*(𝕀*f*{*FC*}) is derived and optimally reconstructed, *I*_*diff*_ on search information is highest across all tasks. However, under full reconstruction *m* = 200 (which is equivalent to using the original functional connectomes), *I*_*diff*_ scores are highest for the functional connectome for all fMRI tasks.

**Figure 5.**
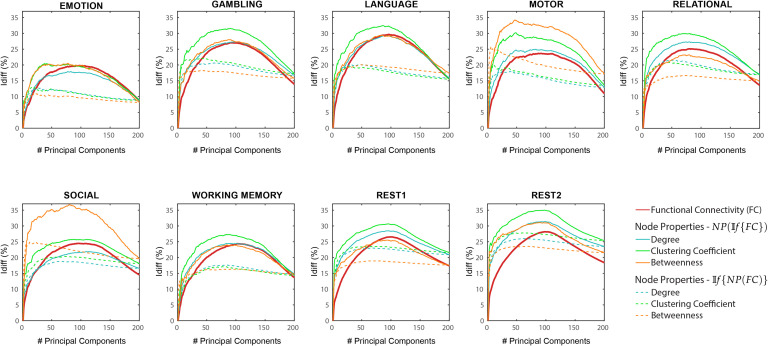
*NP*(𝕀*f*{*FC*}) and 𝕀*f*{*NP*(*FC*)} Differential identifiability (*I*_*diff*_) of node properties for different fMRI tasks as a function of the number of principal components used for reconstruction. Each plot shows, for each task, the *I*_*diff*_ score associated with functional connectivity (red solid line), the *I*_*diff*_ scores on the network properties derived from the reconstructed functional connectomes *NP*(𝕀*f*{*FC*}) (solid lines, colors - see legend) and the *I*_*diff*_ scores on the reconstructed network properties derived from the original functional connectomes 𝕀*f*{*NP*(*FC*)} (dotted lines, colors - see legend) for different numbers of components.

We then assessed how differential identifiability varies based on node properties: degree, betweeness centrality and clustering coefficient (Figure 5). We find that the *I*_*diff*_ profiles of *NP*(𝕀*f*{*FC*} are similar to that of 𝕀*f*{*FC*}. These also give a significantly higher optimal *I*_*diff*_ score for gambling, language, motor, and working memory tasks for all node properties. Especially in the case of language and motor tasks, betweeness centrality gives a significantly higher *I*_*diff*_ of 37 and 35 respectively at optimal reconstruction. For 𝕀*f*{*NP*(*FC*)}, results show lower and flatter *I*_*diff*_ profiles for all tasks and a wide range of number of components. *I*_*diff*_ profiles using *NP*(𝕀*f*{*FC*}) of these node properties are in agreement with all pairwise properties explored so far. In contrast, the *I*_*diff*_ profiles using 𝕀*f*{*NP*(*FC*)} on these node properties are similar to 𝕀*f*{*MFPT*(*FC*)} only.

**Figure 6.**
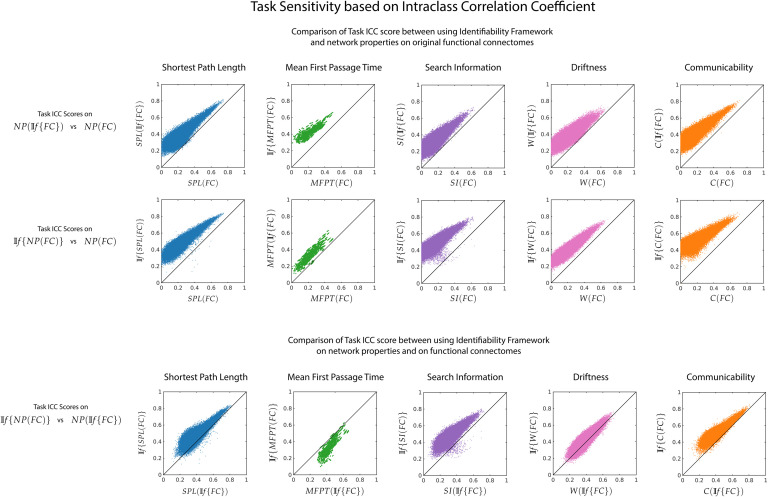
Effect of 𝕀*f* on task sensitivity of network measures. For each pairwise network property, task sensitivity is measured using ICC between *NP*(𝕀*f*{*FC*}) vs *NP*(*FC*) (top row), 𝕀*f*{*NP*(*FC*)} versus *NP*(*FC*) (middle) and *NP*(𝕀*f*{*FC*}) vs 𝕀*f*{*NP*(*FC*)} (bottom row). The first two rows highlight the fact that the 𝕀*f* framework uncovers the inherently distinct signature of different tasks through derived network properties. The last row shows that certain network properties would benefit more from application of the 𝕀*f* framework on the functional connectomes, while others from application directly on the network properties.

Intraclass correlation coefficient was used to assess the task sensitivity of each pairwise network property for three possible cases: *NP*(𝕀*f*{*FC*}) vs *NP*(*FC*) (Figure 6, top row), 𝕀*f*{*NP*(*FC*)} vs *NP*(*FC*) (Figure 6, middle row) and *NP*(𝕀*f*{*FC*}) vs 𝕀*f*{*NP*(*FC*)} (Figure 6, bottom row). We find that the task sensitivity is higher for all network properties when the identifiability framework was used (for both *NP*(𝕀*f*{*FC*}) and 𝕀*f*{*NP*(*FC*)}). Between *NP*(𝕀*f*{*FC*}) and 𝕀*f*{*NP*(*FC*)}, there is no one method that improves task sensitivity for all network properties.

## DISCUSSION

Brain connectivity fingerprinting has taken center stage in the neuroscientific community (Byrge & Kennedy, 2019; Finn et al., 2015; Gratton et al., 2018; Mars et al., 2018; Miranda-Dominguez et al., 2014; Satterthwaite et al., 2018; Seitzman et al., 2019; Venkatesh et al., 2019). As we move in this direction, there is a need to improve the reliability and robustness of individual fingerprint in functional connectomes and on common network measures extracted from functional connectomes. The identifiability framework (𝕀*f*) has shown the capacity to uncover subject fingerprint as measured by the *I*_*diff*_ score in human functional connectomes, regardless of the fMRI task (Amico and Goñi, 2018b). Improving differential identifiability using the 𝕀*f* framework on functional connectomes (FCs) has been shown to improve the test-retest reliability of FCs and correlation with fluid intelligence (Amico and Goñi, 2018b). Here, we extend this framework to show that by maximizing individual fingerprints in the functional connectomes, we also maximize individual fingerprint in network properties derived from the connectomes. Furthermore, we found that uncovering individual fingerprinting on network measurements also improves task signature. In addition, we show that in certain network properties, we can uncover an even stronger fingerprint if we apply the framework directly on the network property instead of functional connectomes.

Numerous work has been done to assess the effect of a change in parameters of the acquisition process and the preprocessing pipelines on test-retest (TRT) reliability of fMRI data (Birn et al., 2013; Noble, Scheinost, & Constable, 2019; Noble et al., 2017; Shah, Cramer, Ferguson, Birn, & Anderson, 2016). The impact of different correlation metrics, inclusion or exclusion of edges on functional connectomes, as well as the use of global signal regression, have been explored extensively (Byrge & Kennedy, 2019; Cao et al., 2014; Fiecas et al., 2013; Liang et al., 2012; Schwarz & McGonigle, 2011; Wang et al., 2011). Additionally, TRT reliability is also seen to be affected by band pass filtering, scan length, sampling rate, network definition of the weights, and size of voxels for node definition (Braun et al., 2012; Liang et al., 2012; Liao et al., 2013). Given that the TRT reliability of the fMRI data and the subsequent estimation of functional connectomes are affected by such diverse factors, it is important to explore the reliability of the derived network properties. Even though TRT reliability is not the only parameter to take into account when choosing the optimal strategy for brain network analyses, it surely has to be considered an important factor to help in such an important choice.

Essentially, 𝕀*f* works as a group-level data-driven (*denoising*) procedure where the components not contributing towards test-retest reliability of FCs are identified and removed. 𝕀*f* doesn’t just improve the overall TRT reliability of a functional connectome but also improves it locally on an edge-level (Amico and Goñi, 2018b) which should ensure that both global and local network properties computed using these denoised functional connectomes are more reliable and robust. As shown in Figure 1, 𝕀*f* not only maximizes subject fingerprint at the FC level, but also at the network property level, which validated our premise. In addition, this convergent behavior is not present just at the optimal point; the identifiability profile of network properties follows the identifiability profile of the functional connectomes. In essence, we have shown that regardless of whether you are using functional connectomes or the network properties derived from them, using 𝕀*f* framework on the functional connectomes would be a beneficial first step.

A natural next question was to find whether 𝕀*f* should be applied on functional connectomes and then derive the network properties (*NP*(𝕀*f*{*FC*})), or to use it directly on the network properties derived from original functional connectomes (𝕀*f*{*NP*(*FC*)}). The two approaches are an attempt to understand different principles of what a fingerprint is in a network derived measurement. 𝕀*f*{*NP*(*FC*)} assumes that functional connectomes are “holding” the individual fingerprints and then propagating them to the network measurements. The fact that maximizing fingerprint of functional connectomes also maximizes the fingerprint in derived network measures, suggests that functional connectomes do indeed hold a subject fingerprint that is then transmitted to the derived network properties. On the other hand, we also see that for some network measures (e.g., search information), we can uncover a better fingerprint if we apply the framework directly on the network measure. This suggests that specific network measures have a subject fingerprint of their own which gets added on to the functional connectome fingerprint. Hence, if under some circumstances, the goal is to maximize the reliability and the individual variability of a specific network property, one can benefit from applying the 𝕀*f* framework on the network property itself, rather than on FCs.

Notably, in the 𝕀*f*{*SI*(*FC*)} scenario, the most different *I*_*diff*_ profiles were found between MFPT and search information (Figure 4). Search information consistently provides a better fingerprint across all tasks than does functional connectome. MFPT, however, can neither improve nor match the fingerprint of functional connectomes. Also, it can not retain the fingerprint that is otherwise present is the functional connectomes and is then propgated to MFPT using 𝕀*f*{*MFPT*(*FC*)}. Hence, while some properties (i.e., search information) can derive higher identifiability than functional connectomes, properties like MFPT need to be computed on optimally reconstructed functional connectomes to uncover subject identifiability on it.

These findings show that brain fingerprinting can be improved by deriving network measurements that extract multivariate information from bivariate measurements such as pairwise correlations used to estimate FCs. Specifically, individual fingerprint peaks on network measurements (e.g., search information) that are more multivariate and requires more information on the global topology of the functional network. However, if the information is heavily driven by degree properties (e.g., MFPT), then there is no improvement on the individual fingerprint (Figure 4). This is strongly corroborated by the *I*_*diff*_ profiles of several node properties under the 𝕀*f*{*NP*(*FC*)} scenario. These profiles are very similar to that of MFPT, a network property which has a strong negative correlation with the degree of the target node. Although 𝕀*f*{*NP*(*FC*)} of these node properties have *I*_*diff*_ profiles similar to 𝕀*f*{*MFPT*(*FC*)}, the maximum *I*_*diff*_ on these node properties are, for some tasks, significantly higher than 𝕀*f*{*FC*}. Betweeness centrality, for example, has a higher subject identifiability for social and motor tasks.

It was interesting to observe that under the 𝕀*f*{*NP*(*FC*)} scenario, betweenness centrality maximizes differential identifiability using just the first two components for social and motor tasks and that it was higher than the identifiability of the functional connectomes for any number of components. Since betweenness centrality can be used to identify integrative communication hubs in FCs (Sporns, 2013), it can be argued that social and motor tasks display a “hub functional fingerprint”, which can be captured by the first two principal components.

A complementary assessment to the identification of subject fingerprints is to assess the ability to identify the different tasks used in this study. To do so, we used intraclass correlation coefficient on the derived network properties. The 𝕀*f* framework improved task sensitivity on the network properties (see Figure 6). Regardless of using the framework on the original functional connectomes or on the network properties themselves, a higher task sensitivity is obtained using one of the process depending on the network property. In both cases, the task reliability of the network properties has improved. The different tasks in the HCP dataset aim to assess different cognitive processes. Hence, the corresponding connectomes and the network properties derived from them should, at least to some extent, be task specific. We have shown that using the 𝕀*f* framework uncovers task-related fingerprints where unique cognitive processes result in differential network properties.

To summarize, differential identifiability was found to be always higher on functional connectomes than on any network properties when the identifiability framework (𝕀*f*) is not used. When 𝕀*f* improved identifiability on functional connectomes, the identifiability on the network properties also increased. The framework also improved the subject fingerprints of the network properties. Not only do they improve at the optimal point, but the differential identifiability follows the same profile on network properties as it does on functional connectomes. We also find that applying the identifiability framework on the network properties instead of functional connectomes gives higher differential identifiability for some network properties. At optimal reconstruction, we find that search information has higher differential identifiability than functional connectomes across all tasks when the identifiability framework is applied on search information. This shows that there are network properties that can uncover better identifiability with the framework than the functional connectomes themselves. Finally, we found that using the identifiability framework (either on functional connectomes or network property) improves task sensitivity in all network properties.

Our study has some limitations. Only the unrelated subjects of the Human Connectome project and the cortical parcellation proposed by Glasser et al. (2013) are used in this work. Other explorations with other atlases, parcellations and/or other estimators of functional coupling (other than Pearson’s correlation coefficient) would expand on the implications of our work. We have also limited our study to commonly used five pairwise and three node network properties. Delving into other network properties can strengthen this framework further and provide additional insights in understanding the associations between brain fingerprints, functional connectivity, and network derived properties. It could also be possible that relevant combinations of network measurements (driftness is an example of it) might uncover additional brain fingerprints and reach even higher differential identifiability levels.

This study can be extended to clinical applications to understand diseases that target specific functions of the human brain. For instance, for assessing pathologies whose signature cannot be mapped on the functional connectomes themselves but can be assessed using different network properties derived from them (Bassett & Bullmore, 2009; Fornito & Bullmore, 2015; Fornito et al., 2015) In this case, to retain individual differences and to be able to differentiate healthy population from clinical ones, we need this study to understand the advantages of using the identifiability framework on the functional connectome or network property. Finally, studying the effect of the framework on the structural connectome is another natural extension of this work.

## ACKNOWLEDGMENTS

Data were provided (in part) by the Human Connectome Project, WU-Minn Consortium (principal investigators: David Van Essen and Kamil Ugurbil; 1U54MH091657), funded by the 16 NIH Institutes and Centers that support the NIH Blueprint for Neuroscience Research; and by the McDonnell Center for Systems Neuroscience at Washington University. The authors thank Dr. Gorka Zamora-Lopez and Dr. Matthieu Gilson for useful comments.

## AUTHOR CONTRIBUTIONS

Meenusree Rajapandian: Conceptualization; Data curation; Formal analysis; Visualization; Writing - Original Draft. Enrico Amico: Data curation; Methodology; Writing - Original Draft. Kausar Abbas: Formal analysis; Methodology; Writing - Original Draft. Mario Ventresca: Supervision; Writing - Original Draft. Joaqun Goi: Conceptualization; Data curation; Formal analysis; Funding acquisition; Methodology; Resources; Supervision; Writing - Original Draft.

## FUNDING INFORMATION

Joaquín Goi, National Institutes of Health (U. S.), Award ID: R01EB022574. Joaqun Goi, National Institutes of Health, Award ID: R01MH108467. Joaqun Goi, National Institutes of Health, Indiana Alcohol Research Center, Award ID: P60AA07611. Joaqun Goi and Mario Ventresca, Purdue Discovery Park Data Science Award “Fingerprints of the Human Brain: A Data Science Perspective.”

## TECHNICAL TERMS

Principal component analysis (PCA): A dimensionality reduction technique that uses an orthogonal transformation to convert a set of observations of (possibly) correlated variables into a set of values of linearly uncorrelated variables called principal components. Such transformation ensures that the first principal component has the largest possible variance, and each subsequent component has the highest possible variance under the constraint of being orthogonal to the preceding components.
Differential identifiability: A score that quantifies in a test/retest dataset, on average, how much more similar are rest/retest functional connectomes of the same subject with respect to functional connectomes of different subjects. Similarity is measured by Pearsons correlation coefficient between every two functional connectivity profiles.
Functional magnetic resonance imaging (fMRI): A noninvasive technique that estimates brain activity by detecting changes associated with blood flow. The rationale of this technique relies on the fact that there is a positive association between cerebral blood flow and neuronal activation.
Identifiability framework: A framework based on principal component analysis to decompose a test/retest functional connectivity dataset (or its network-derived measurements) and subsequently reconstruct at the optimal number of components that maximizes the differential identifiability score.
Functional connectome/connectivity (FC) matrix: A network representation of the functional coupling between brain regions. Such coupling is usually measured by quantifying the statistical dependencies between timeseries of brain regions (e.g., pairwise Pearson’s correlation, mutual information) as obtained by fMRI.
Graph: An ordered pair formed by a set of nodes and a set of edges (which represent connections between pairs of nodes). Nodes are usually represented by circles, whereas edges are represented by lines or arcs connecting pairs of nodes.
Mean first passage time (MFPT): In a connected graph, MFPT quantifies the expected number of steps that it takes for a random walker to go from a source node to a target node for the very first time. The measurement relies on the transition probability matrix that can be obtained from a connected graph, which is indeed an ergodic Markov chain.
Node strength: In a weighted graph (i.e., where edges have assigned numerical values), it represents the total sum of the edge weights attached to the node.
Search information (SI): An information theoretical measurement that quantifies in bits how hidden a shortest path is (from a source node to a target node), as embedded in the graph. This measurement may be applied to binary and weighted graphs.
Intraclass correlation (ICC): An inferential statistic for quantitative measurements that are organized into groups. ICC describes how strongly units in the same group resemble each other. A typical application consists of the assessment of consistency or reproducibility of quantitative measurements made by different observers measuring the same quantity.
